# A rare case report of multifocal para-aortic and para-vesical paragangliomas

**DOI:** 10.3389/fendo.2022.946496

**Published:** 2022-08-08

**Authors:** San-Chao Xiong, Xing-Peng Di, Meng-Ni Zhang, Kan Wu, Xiang Li

**Affiliations:** ^1^ Department of Urology, Chengdu Second People’s Hospital, Chengdu, China; ^2^ Department of Urology, Institute of Urology, West China Hospital, Sichuan University, Chengdu, China; ^3^ Department of Pathology, Institute of Pathology, West China Hospital, Sichuan University, Chengdu, China

**Keywords:** paraganglioma, case report, MIBG, surgery, catecholamine

## Abstract

**Background:**

Paragangliomas (PGLs) are uncommon tumors of uncertain malignant potential. Multifocal paragangliomas are scarcely reported in the literature.

**Case summary:**

A 25-year-old male patient was reported for the first time with multifocal para-aortic and para-vesical PGLs. The diagnosis was identified by blood catecholamine tests and enhanced CT scan and MIBG scintigraphy. A resection surgery was performed for treatment and the immunochemistry test of the tumors presented the features of PGL.

**Conclusion:**

A case of multifocal para-aortic and para-vesical PGLs confirmed by the catecholamine test, enhanced CT, and MIBG scintigraphy is presented. The cooperation of experienced surgeons, anesthesiologists, and endocrinologists was critical in treatment.

## Introduction

Paragangliomas (PGLs) are uncommon tumors consisting of neural crest cells that originate from the paravertebral sympathetic chains and parasympathetic ganglions of the chest, abdomen, and pelvic cavity (1). The incidence of PGLs is three to eight cases per 1,000,000 person-years (2). PGLs are extra-adrenal pheochromocytomas. They are derived from neuroendocrine chromaffin cells (3). Since the fourth edition of the WHO, PGLs have no longer been classified as benign or malignant, as any lesion can have metastatic potential and there are no clear-cut features that can predict metastatic behavior.

Most PGLs secrete catecholamines and present with episodes of high blood pressure, heart palpitations, sweating, and headache. Some may be hormonally inactive, but this is the minority. In such cases, presentation will be due to pain and functional loss due to invasion into neighboring structures and organs. However, much more commonly, many of these tumors are diagnosed incidentally because of medical imaging for other reasons (4). The metastatic and regional infiltrated PGLs may lead to devastating and irreversible consequences (5). Moreover, 40% of the PGL patients suffer from a recurrence of malignancy within 5 years of surgery (6).

Here, we present a case report of a patient with multifocal para-aortic para-vesical PGLs.

## Case conundrum

A 25-year-old male was admitted to the Urology Ward of West China Hospital, Sichuan University, in March 2022. One month ago, a tumor close to the urinary bladder was incidentally found during ultrasonography in routine physical examination. The records at admission showed that the blood pressure of the patient was 126/73 mmHg, and the heart rate was 80 bpm when he was inpatient. No other chief complaints or marked symptoms were presented, such as headaches, blood pressure turbulence, or abdominal pain. The patient had no history of hepatitis and tuberculosis, nor did he have a history of allergies, trauma, surgery, familial disease, alcoholism, or smoking. The physical examination showed a soft abdomen, and no mass, regional lymphadenopathy, or pain was reported.

**Figure 1:**
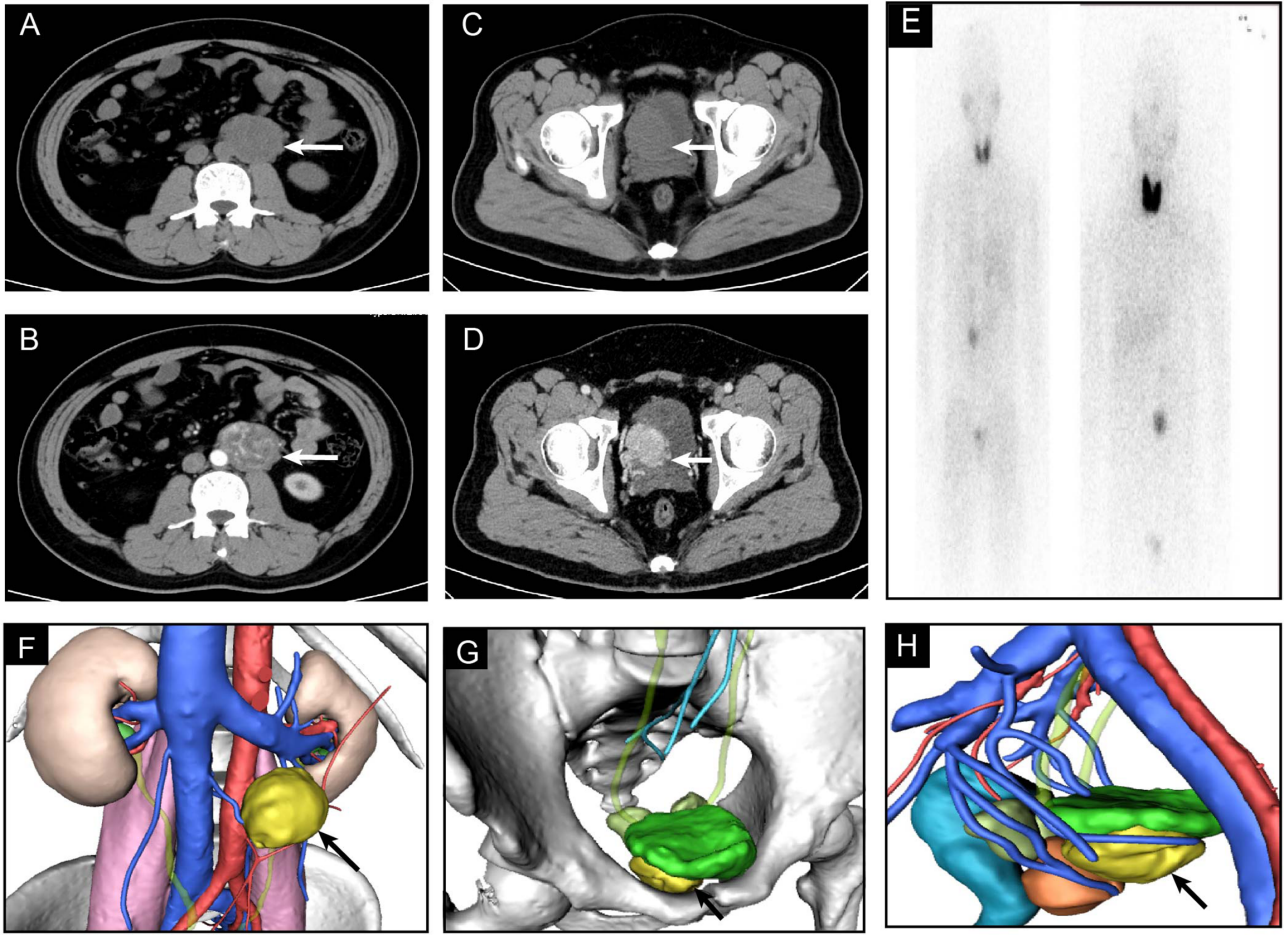
The enhanced CT and 3D model of PGLs. **(A, B)** Plain and enhanced CT images of PGL in abdomen. **(C, D)** Plain and enhanced CT images of PGL in pelvis. **(E)** MIBG scintigraphy. **(F–H)** 3D model of CT imaging.

An abdominal CT scan showed an additional left para-aortic tumor with enhanced density (**Figure 1**). The ^131^I-mataiodobenzyl-guanidine (MIBG) scintigraphy results showed two radio-uptake enhancing regions in the left abdomen and pelvis after 48 and 72 h (**Figure 1E**). Laboratory tests showed an increase in the level of norepinephrine (10.37 nmol/L), normetanephrine (7.01 nmol/L), and 3-methoxytyramine (23.97 nmol/L). The characteristics, diagnosis, and therapies of the disease were discussed. The patient presented with normal blood pressure and heart rate. However, significantly increased serum catecholamines were detected. A preliminary diagnosis of multifocal PGLs was confirmed by an additional metaiodobenzylguanidine (MIBG) scan.

Laparoscopic resection of the tumor adjacent to the bladder wall and the para-aortic tumor was recommended. In order to avoid hypotensive episodes during surgery, the patient was treated with pre-operative adrenergic blockade (10 mg of the α adrenoreceptor blocker phenoxybenzamine). The tumor adjacent to the bladder measured 4.0 × 3.1 × 2.5 cm, and the para-aortic tumor measured 5.6 × 5.2 × 4.6 cm. The patient’s postoperative course was uneventful and the patient was discharged 4 days post-surgery.

**Figure 2:**
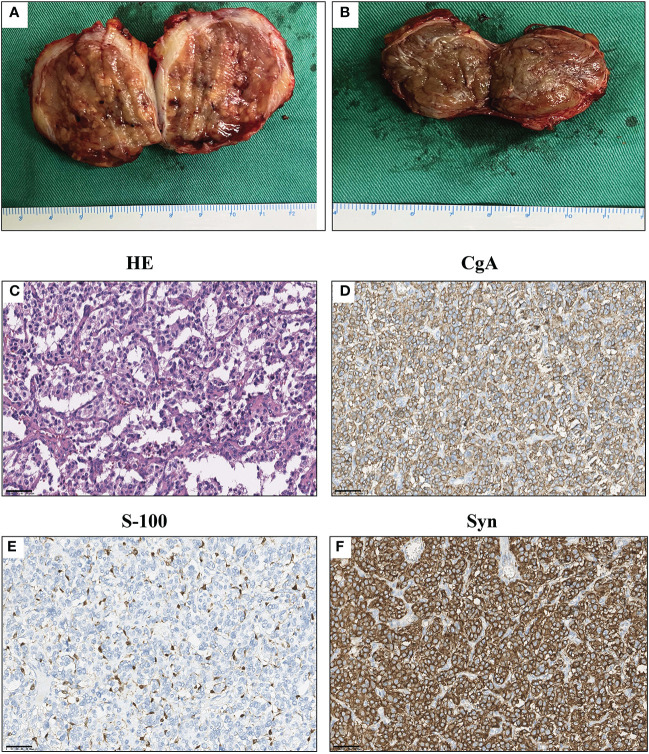
Macroscopic examination and immunochemistry results of the tumors. **(A)** Macroscopic examination of para-aortic PGL. **(B)** Macroscopic examination of para-vesical PGL. **(C)** HE staining of tumors. **(D–F)** Immunohistochemistry results of the tumors. Syn (+), CgA (+), S-100 (+), and the Ki-67 positive index was 1%. Scale bar = 50 μm.

The final diagnosis was confirmed by histopathology of the specimens: The envelopes of the tumors were intact; the cystic-solid tumor close to the aorta presented a grey-white nodular morphology with hemorrhage and necrosis, cysts were individual and smooth, with yellow-brown tissues inside (**Figure 2A**); and the other nodular tumor showed a similar morphology (**Figure 2B**). Further immunochemistry results reported Syn (+), CgA (+), S-100 (+), GATA3 (+), PCK (-), HMB45 (-), and MART-1 (-), and the Ki-67 positive index was 1% (**Figures 2C–F**). Combined with the morphological features, the tumors were considered as PGLs. The patient’s consent has been obtained before the acquisition of relevant data and materials.

## Discussion

PGLs are rare tumors that originated from the neural crest, consisting of 17% of primary extra-adrenal tumors (7). Approximately 3% of PGLs were reported as multifocal tumors (8). The PGLs produce one or more kinds of catecholamines, including epinephrine, norepinephrine, and dopamine. Excessive secretion of catecholamines results in hypertension, tachycardia, headache, and sweating (9). Blood pressure must be controlled before the surgery with alpha blockade since prolonged exposure to excessive catecholamines can lead to a hypotensive crisis during resection of these tumors (10).

PGLs were commonly diagnosed by symptoms such as high blood pressure, heart palpitations, sweating, and headache with blood catecholamine increase, CT scan, and MIBG scintigraphy. It was reported that about 89%–95% of the extra-adrenal PGLs were observed in the abdomen or pelvis, of which 75% were observed in para-aortic areas and 10% were observed in the urinary bladder (11). As one of the most common symptoms of PGLs, hypertension was reported in 51%–90% of patients with pheochromocytomas (12, 13). However, in the current case, no marked symptom was observed. The CT imaging of PGLs may be homogeneous and heterogeneous, either solid or cystic. Some PGLs are presented with calcifications. The PGLs in the case above were all solid-cystic tumors without calcification. Alternative examinations for diagnosis include MRI and functional imaging. Radiomic-based nomograms, such as MIBG scintigraphy, increase the accuracy of the predictive value of PGLs (14). MIBG accumulates in catecholamine-producing cells, and ^123^I-labeled MIBG assists in the detection of PGLs in a highly sensitive manner (56%–75%) (15). The MIBG imaging also delivered a satisfying result in this case.

The pathology mechanism of PGLs varies. One such mechanism is that the Nfr2 activation and the elevated glucose uptake contribute to PGL malignancy with SDHB gene mutation (16). VHL and MEN-2 have a low malignancy risk of PGLs. In sporadic PGL, succinyl-CoA G2 protein was identified as a novel candidate gene through genetic testing (17).

In conclusion, this case showed that the diagnosis of PGLs can be confirmed by the catecholamine test, enhanced CT, and MIBG scintigraphy. Despite the absence of hypertension in patients, a pre-operative adrenergic blockade is of great importance (18). Surgery with the cooperation of experienced surgeons, anesthesiologists, and endocrinologists is the first line of treatment for PGLs. For irresectable lesions, ^131^I-MIBG therapy is well adopted in PGL patients and reduces the catecholamine levels (19). Anti-neoplastic chemotherapy and radiotherapy are also treatments for PGLs with a malignancy potential (20). Although PGL is a benign tumor, the malignant tendency of masses still requires long-time follow-ups.

## Data availability statement

The original contributions presented in the study are included in the article/**Supplementary Material**. Further inquiries can be directed to the corresponding author.

## Ethics statement

Ethical review and approval was not required for the study on human participants in accordance with the local legislation and institutional requirements. The patients/participants provided their written informed consent to participate in this study. Written informed consent was obtained from the individual(s) for the publication of any potentially identifiable images or data included in this article.

## Author contributions

Conceptualization: XL. Data curation and Project administration: S-CX and KW. Staining slides provision: M-NZ. Manuscript Writing—Original draft: S-CX and X-PD. Manuscript editing and manuscript review: XL and X-PD. This manuscript has been read and approved by all the authors.

## Funding

This work was supported by the Sichuan Science and Technology Program (2022YFS0133).

## Conflict of interest

The authors declare that the research was conducted in the absence of any commercial or financial relationships that could be construed as a potential conflict of interest.

## Publisher’s note

All claims expressed in this article are solely those of the authors and do not necessarily represent those of their affiliated organizations, or those of the publisher, the editors and the reviewers. Any product that may be evaluated in this article, or claim that may be made by its manufacturer, is not guaranteed or endorsed by the publisher.
