# Supplementary material for: A rare case report of multifocal para-aortic and para-vesical paragangliomas
Source: Front Endocrinol (Lausanne). 2022 Aug 8;13:946496. doi: 10.3389/fendo.2022.946496 (PMC9393499; doi:10.3389/fendo.2022.946496)
Supplement: Supplementary file 1 [file Table_1.docx]

| **Items** | **Results** | **Unit** | **Reference** |
| --- | --- | --- | --- |
| Norepinephrine (NE) | 10.37 | nmol/L | <5.17 |
| Methoxynorepinephrine (NMN) | 7.01 | nmol/L | <0.71 |
| 3-Methoxytyramine (3-MT) | 23.97 | pg/ml | <18.40 |
| adrenocorticotropic hormone (ACTH) | 31.13 | ng/L | 5.00-78.00 |
| Epinephrine (E) | 0.21 | nmol/L | <0.34 |
| Dopamine (DA) | 0.2 | nmol/L | <0.31 |
| Metanephrine (MN) | 0.21 | nmol/L | <0.42 |
| Critisol (PTC) | 269 | nmol/L | — |
| Total bilirubin | 15.1 | μmol/L | 5.0-28.0 |
| Direct bilirubin | 5.4 | μmol/L | <8.8 |
| Alanine aminotransferase | 97 | IU/L | <50 |
| Aspartate aminotransferase | 32 | IU/L | <40 |
| Total protein | 78.2 | g/L | 65.0-85.0 |
| Albumin | 47 | g/L | 40.0-55.0 |
| Glucose | 4.8 | mmol/L | 3.90-5.90 |
| Creatinine | 74 | μmol/L | 68.0-108.0 |
| Cholesterol | 3.7 | mmol/L | 2.80-5.70 |
| Red blood cell | 5.37 | 1012/L | 4.3-5.8 |
| White blood cell | 8.03 | 109/L | 3.5-9.5 |
| Platelet | 271 | 109/L | 100-300 |

**Supplementary Material**

**Table.** The results of laboratory exams.
